# *Notes from the Field:* Community Outbreak of Measles — Clark County, Washington, 2018–2019

**DOI:** 10.15585/mmwr.mm6819a5

**Published:** 2019-05-17

**Authors:** Alyssa Carlson, Madison Riethman, Paul Gastañaduy, Adria Lee, Jessica Leung, Michelle Holshue, Chas DeBolt, Alan Melnick

**Affiliations:** ^1^Clark County Public Health, Vancouver, Washington; ^2^Division of Viral Diseases, National Center for Immunization and Respiratory Diseases, CDC; ^3^Epidemic Intelligence Service, CDC; ^4^Office of Communicable Disease Epidemiology, Washington State Department of Health.

On December 31, 2018, Clark County Public Health (CCPH) in Washington was notified of a suspected case of measles in an unvaccinated child, aged 10 years, who had recently arrived from Ukraine. The patient was evaluated at an urgent care clinic for fever, cough, and a maculopapular rash. CCPH launched a case investigation, conducted contact tracing, and facilitated specimen collection and shipment to the Washington State Department of Health Public Health Laboratories. On January 3, 2019, measles virus was detected in the patient’s urine and nasopharyngeal specimens by reverse transcription–polymerase chain reaction (RT-PCR). By January 16, among 12 patients with suspected measles reported to CCPH during January 11–14, all had laboratory-confirmed measles by RT-PCR. In response to these confirmed cases and additional suspected cases, CCPH’s Incident Management Team was activated on January 15. Approximately 200 persons participated in the multiagency response, which included CCPH, the Washington State Department of Health, and CDC. As of March 28, 2019, measles had been confirmed among 71 Clark County residents, with rash onsets from December 30, 2018, to March 13, 2019.

Persons with suspected measles were investigated through patient interviews, electronic medical records review, and consultation with health care providers; specimens were collected in accordance with recommendations (*1*). To increase awareness of measles circulation, regional provider advisories were issued, and press releases were distributed to notify citizens of exposures in large settings. Outbreak control measures included identifying exposed persons and assessment of their presumptive immunity to measles,* recommending vaccination of persons lacking presumptive evidence of immunity, administering postexposure prophylaxis with measles, mumps, rubella vaccine or immunoglobulin for eligible persons, and implementing social distancing strategies (e.g., isolation of patients and home quarantine of exposed persons without presumptive evidence of immunity) (*2*).

Among the 71 patients with confirmed measles, all of whom met the clinical case definition for measles,^†^ 41 cases were laboratory-confirmed and 30 were epidemiologically linked to confirmed cases (*3*). Patients were aged 1–39 years (median = 8 years); 52 (73%) were children aged ≤10 years. Sixty-one (86%) were unvaccinated, three (4%) had received 1 dose of measles, mumps, rubella vaccine before measles exposure, and vaccination status was unknown for seven (10%). Genotype D8, which is currently circulating in Eastern Europe, was identified in all 18 specimens tested (*4*). No new confirmed cases have been identified since March 13, 2019.

Approximately 3,800 named contacts of the 71 patients were identified from 46 known exposures at Clark County health care facilities, workplaces, churches, schools, and child care centers, as well as social gatherings and home settings. Among these contacts, 22% lacked acceptable presumptive evidence of measles immunity.

Households and churches were the predominant settings for transmission, associated with 36 (51%) and 18 (25%) of the 71 patients, respectively (Figure). Public exposures (i.e., church, school, and child care centers) most commonly occurred during the first 4 weeks of the outbreak, and decreased following communitywide implementation of CCPH-recommended outbreak control measures. Among the 30 patients identified after February 1, 26 (87%) were known contacts in quarantine and under active surveillance, decreasing public exposures by implementing effective social distancing strategies.

**FIGURE:**
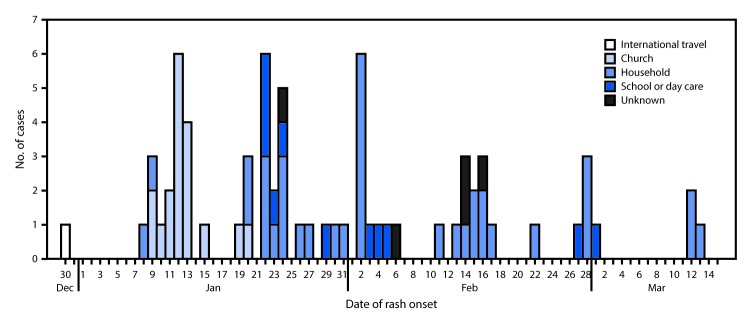
Number of measles cases, by transmission setting and date of rash onset (N = 71) — Clark County, Washington, December 30, 2018–March 13, 2019

Clark County had not experienced a measles outbreak since 2011, when three cases were confirmed (*5*). Since 2013, county vaccination rates have remained 10%–14% lower than the statewide average (88%) (*6*). Additional U.S. jurisdictions are experiencing concurrent, unrelated measles outbreaks (*7*). As of May 10, 2019, the 839 measles cases identified in 23 states nationwide had surpassed the case counts observed during the same period every year since 2000, when measles was declared eliminated in the United States (range = 6–164; CDC, unpublished data). The current U.S. outbreaks underscore the importance of maintaining 2-dose measles vaccination coverage of ≥95% and of rapid public health responses in an era of increasing measles exposure threat, both in the United States and around the world.

## Footnotes

* Acceptable presumptive evidence of immunity to measles includes written documentation of age-appropriate vaccination, laboratory evidence of immunity, laboratory confirmation of disease, or birth before 1957.

† Fever; maculopapular rash; and cough, coryza, or conjunctivitis.
